# Oesterlen's Handbook of Hygiene
*Handbuch der Hygieine, der privaten und öffentlichen. Von F. Oesterlen, M. Dr. pp. 837. Tübingen: 1857.


**Published:** 1857-10

**Authors:** 


### THE EPITOME OF SANITARY LITERATURE.

### OESTERLEN'S HANDBOOK OF HYGIENE.*

PROFESSOR OESTERLEN'S *Handbook* is rather a serious volume of 837 8vo pages in compact type. The subjects of the chapters are: I. Man in his changing Conditions and Necessities: II. The Atmosphere and Meteorological Influences: III. Water and Hydrological Influences: IV. Soil and Telluric Influences: V. Isolated Regions and Topography: VI. Climate: VII. Foods and Drinks: VIII. Dwellings and Public Buildings: IX. Dress, Washing, and Bathing: X. The Generic Functions and Relations: XI. Exercise and Training of the Body: XII. Occupations. The whole concludes with an appendix, in which various questions relating to the duration of life are discussed.

We give thus the outline of a very comprehensive work, which offers but few points to discussion, because it is common sense to a fault, and a stronghold of the facts on which the sanitarian builds his faith. It will gratify English pride to hear that Professor Oesterlen looks on England, not only as one of the purest representations of a free country, but as infinitely more enlightened than other European countries in regard to sanitary science. It will gratify smokers to know that the learned Professor has a good word for tobacco. But it will not gratify the ladies to hear him say that the only real cosmetics are, care of the health, temperance, nourishing food, good digestion, care of the skin, and moderate labour; and that anything else is deception and error. Some of the greasy preparations, he says, may be useful to those living in the north and in the tropics, especially for rubbing into their bodies, to soften and preserve their skin from the effects of the heat and cold, dry air, insects, flies, gnats, etc.; but above all, cleanliness is the best cosmetic.

Simple washing powders may, however, be sometimes substituted for soaps, especially for the fair; such powders for instance as rice-meal, almond-bran, and its extract; wheaten bread, soaked in rain water, or soap, first cut small, then dried in the oven, powdered and mixed with equal parts of wheat meal. The face may also be rubbed over in the evening with linden blossom water, mixed with yolk of egg; a cream of peeled and bruised bitter almond or peach kernels with milk, milk of almonds, and some drops of tincture of benzoin: in the morning this may be washed off again, according to the fashion

* Handbuch der Hygieine, der privaten und öffentlichen. Von F. OESTERLEN, M. Dr. pp. 837. Tübingen: 1857.

of the ancient Roman ladies, with bread crumbs and asses' milk. These cosmetics are in no way injurious to health; but corroding poisonous preparations of metals, above all, arsenic, quicksilver, lead, and mineral acids, like those recommended and sold as cosmetics in the present day, are to be entirely avoided.

